# Cellular Prion Protein Expression Is Not Regulated by the Alzheimer's Amyloid Precursor Protein Intracellular Domain

**DOI:** 10.1371/journal.pone.0031754

**Published:** 2012-02-21

**Authors:** Victoria Lewis, Isobel J. Whitehouse, Herbert Baybutt, Jean C. Manson, Steven J. Collins, Nigel M. Hooper

**Affiliations:** 1 Institute of Molecular and Cellular Biology, Faculty of Biological Sciences, University of Leeds, Leeds, United Kingdom; 2 Department of Pathology, University of Melbourne, Parkville, Victoria, Australia; 3 Neuropathogenesis Unit, Roslin Institute, University of Edinburgh, Midlothian, United Kingdom; INSERM, UMR-S747, France

## Abstract

There is increasing evidence of molecular and cellular links between Alzheimer's disease (AD) and prion diseases. The cellular prion protein, PrP^C^, modulates the post-translational processing of the AD amyloid precursor protein (APP), through its inhibition of the β-secretase BACE1, and oligomers of amyloid-β bind to PrP^C^ which may mediate amyloid-β neurotoxicity. In addition, the APP intracellular domain (AICD), which acts as a transcriptional regulator, has been reported to control the expression of PrP^C^. Through the use of transgenic mice, cell culture models and manipulation of APP expression and processing, this study aimed to clarify the role of AICD in regulating PrP^C^. Over-expression of the three major isoforms of human APP (APP_695_, APP_751_ and APP_770_) in cultured neuronal and non-neuronal cells had no effect on the level of endogenous PrP^C^. Furthermore, analysis of brain tissue from transgenic mice over-expressing either wild type or familial AD associated mutant human APP revealed unaltered PrP^C^ levels. Knockdown of endogenous APP expression in cells by siRNA or inhibition of γ-secretase activity also had no effect on PrP^C^ levels. Overall, we did not detect any significant difference in the expression of PrP^C^ in any of the cell or animal-based paradigms considered, indicating that the control of cellular PrP^C^ levels by AICD is not as straightforward as previously suggested.

## Introduction

Alzheimer's disease (AD) and prion diseases fall within the spectrum of neurodegenerative diseases which are causally linked to misfolded and aggregated proteins. Due to similarities in various structural elements and proteolytic processing events involving the major proteins involved in these diseases, potential links and parallels in both disease mechanisms and possible therapeutic avenues have been proposed [1], [2], [3], [4]. Increasingly, recent studies have shown more direct molecular links between AD and prion diseases, and the proteins at the centre of these diseases; namely the amyloid precursor protein (APP) and its proteolytic cleavage product the amyloid-β (Aβ) peptide which deposits as plaques in the AD brain, and the normal cellular prion protein (PrP^C^) and the disease-associated isoform PrP^Sc^, which accumulates in prion diseases. A substantive molecular link was provided when PrP^C^ was shown to modulate production of Aβ from wild type APP, through an interaction with the β-secretase BACE1 [5], later demonstrated to be a mechanism for altered trafficking and localisation of BACE1 resulting in reduced Aβ production [6]. Additionally, several groups have now presented evidence that PrP^C^ can bind oligomeric forms of Aβ [7], [8], [9], [10], although there is conflicting data regarding the downstream consequences of this binding. Some results suggest that Aβ oligomer synaptic toxicity is mediated through its binding to PrP^C^ [7], [11], [12], whereas others have reported that Aβ oligomer neurotoxicity is independent of PrP^C^ expression [8], [9]. Whilst perhaps explained by methodological differences, these opposing results underscore the complexity in the possible interactions between these two key proteins and diseases.

In addition to Aβ, a number of other proteolytic fragments are generated from APP. Cleavage of the full length APP by either α-secretase or BACE1 produces large soluble N-terminal ectodomains, and C-termimal membrane-bound stubs, denoted C83 and C99, respectively. Both C83 and C99 can be cleaved by the γ-secretase complex to produce the APP intracellular domain (AICD) [13]. This latter fragment appears to act as a transcriptional regulator after forming a complex with Fe65 and Tip60 [14]. In particular AICD has been shown to regulate the expression of the Aβ degrading enzyme neprilysin [15], [16]. Interestingly, it appears to be only the AICD produced from the combined action of BACE1 and γ-secretase on APP that is transcriptionally active [17], [18], [19].

There are three major isoforms of APP expressed in the brain, APP_695_, APP_751_ and APP_770_, which are produced via alternative splicing of the single mRNA [20]. Of the three, APP_695_ is the major neuronal splice variant. Recently, we reported that only the AICD produced from the β- and γ-secretase cleavage of APP_695_, and not that produced from the other two isoforms, is transciptionally active as assessed by its ability to upregulate neprilysin expression [19]. This transcriptionally active AICD was only produced in neuronal (SH-SY5Y and N2a) cell lines and was not functional in non-neuronal human embryonic kidney (HEK293) cells [19]. Further, AICD produced from the familial AD associated Swedish mutant form of APP_695_, known to be subject to increased BACE1 cleavage compared to wild type APP_695_ [21], was more transcriptionally active relative to wild type APP_695_ [19].

The molecular and cellular links between APP and PrP^C^ were extended recently when PrP^C^ expression was reported to be regulated by AICD [22]. Overexpression of APP_751_ in HEK cells triggered a significant increase in PrP^C^ immunoreactivity, while a reduction in PrP^C^ was observed in APP deficient fibroblasts. The γ-secretase inhibitor DAPT significantly reduced PrPC levels in primary neurons, implicating a role for AICD in controlling the expression of PrPC [22]. The aim of the present study was to clarify the role of AICD in the regulation of PrP^C^ and to specifically determine whether, similar to the control of neprilysin expression [19], there was an APP isoform effect.

## Results

### Over-expression of APP does not alter endogenous PrP^C^ protein expression

**Figure 1:**
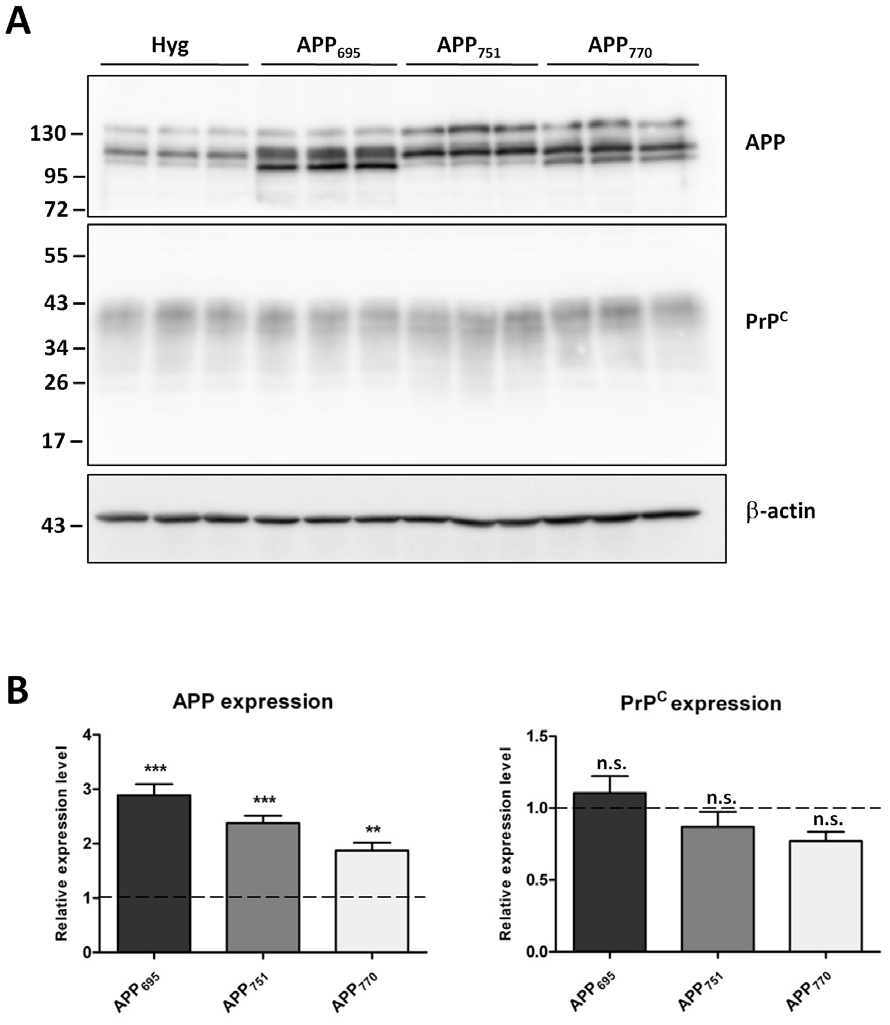
Over-expression of APP isoforms in HEK cells does not alter endogenous PrP^C^. (A) Representative western blot of APP and PrP^C^ (antibody 3F4) in HEK cells stably transfected with either the vector alone (Hyg) or one of the APP isoforms (APP_695_, APP_751_, APP_770_), and subsequent β-actin staining to allow adjustments for equal protein loading. Approximate molecular weights (kDa) are indicated. (B) Quantification of APP and PrP^C^ protein levels expressed relative to Hyg control cells (dashed line). Data from 4 independent experiments. Statistical analysis by one way ANOVA with Dunnett's post test comparison to the Hyg cells, ***p<0.001, **p<0.01, n.s. not significant.

Initially we sought to replicate the findings of Vincent et al. [22] by expressing APP_751_ in HEK cells. In addition, we looked to advance this research by determining whether the control of PrP^C^ expression by AICD was specific to a particular APP isoform. HEK cells stably over-expressing either APP_695_, APP_751_ or APP_770_, alongside a vector only control were assessed for total cell associated PrP^C^ and APP protein levels by western blotting (Fig. 1A and B). Surprisingly, in contrast to previously published results [22], although there was a significant 2–3-fold increase in APP in the cells transfected with any of the three APP isoforms, there was no significant difference in PrP^C^ level in any of the APP isoform expressing cells when compared to the Hyg vector-only controls.

**Figure 2:**
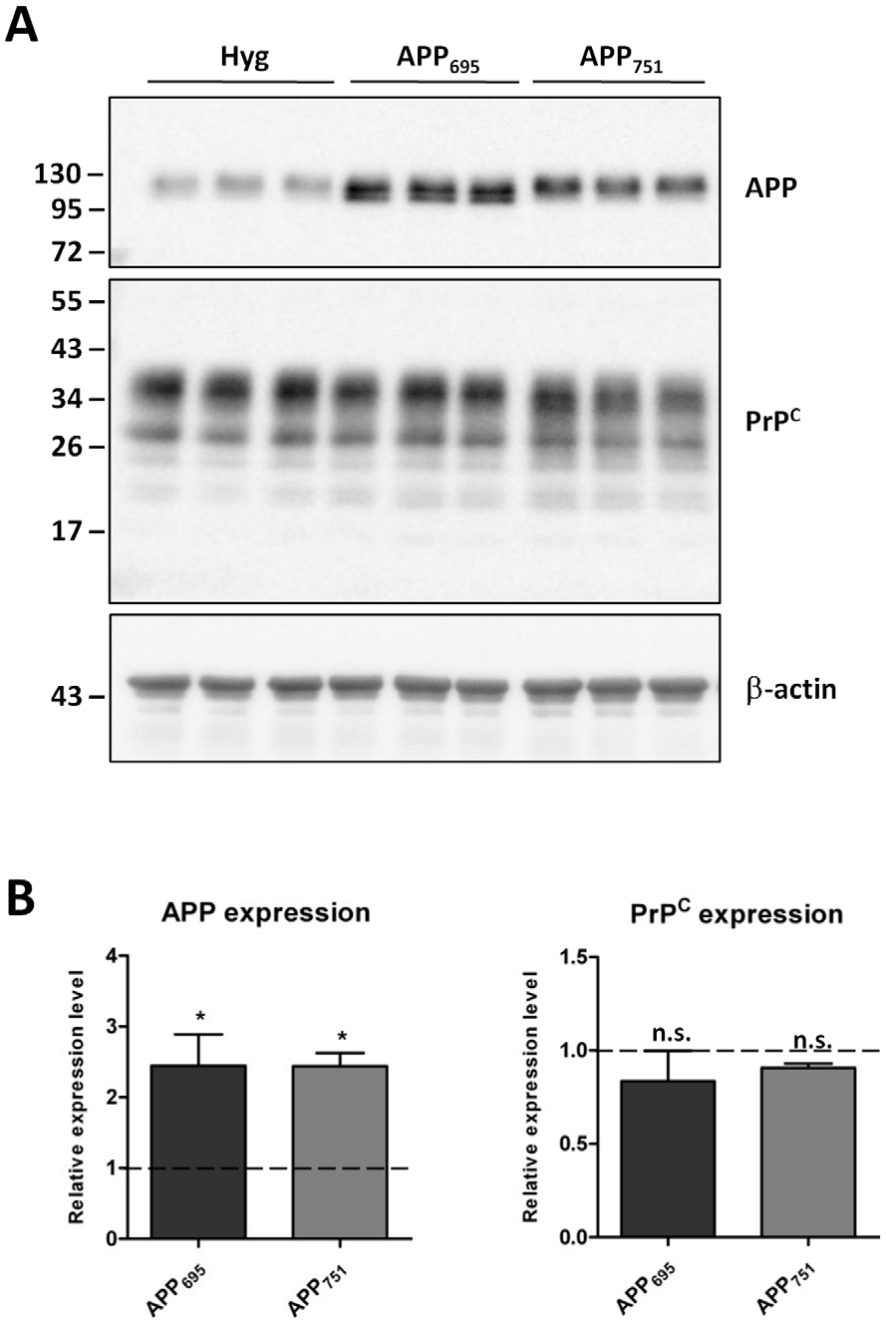
Over-expression of APP isoforms in N2a cells does not alter endogenous PrP^C^. (A) Representative western blot of APP and PrP^C^ (antibody 6H4) in N2a cells stably transfected with either the vector alone (Hyg) or one of the APP isoforms (APP_695_, APP_751_), and subsequent β-actin staining. Approximate molecular weights (kDa) are indicated. (B) Quantification of APP and PrP^C^ protein levels expressed relative to Hyg control cells (dashed line). Data from 3 independent experiments. Statistical analysis by one way ANOVA with Dunnett's post test comparison to the Hyg cells, *p<0.05, n.s. not significant.

We have recently shown that transcriptionally active AICD is only produced by the BACE1 and γ-secretase cleavage of the APP_695_ isoform in neuronal cells [19]. In light of this, and the negative result observed in the non-neuronal HEK cells, we utilized mouse neuronal N2a cells over-expressing human APP_695_ or APP_751_ to again assess total PrP^C^ and APP protein levels (Fig. 2A and B). Despite a significant 2.5-fold increase in APP expression in the N2a cells transfected with the cDNAs encoding either APP_695_ or APP_751_, there was no difference in endogenous PrP^C^ levels when comparing the APP over-expressing cells with each other or the vector only controls.

**Figure 3:**
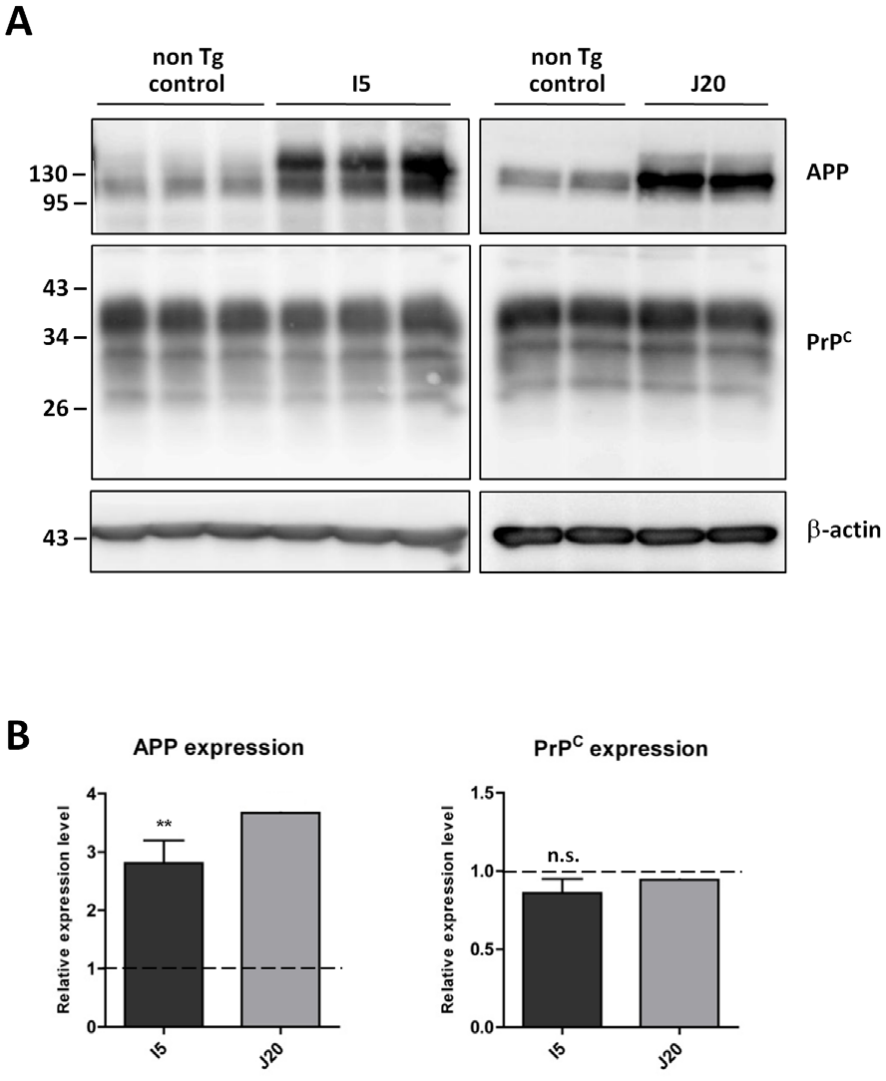
Unaltered PrP^C^ protein levels in transgenic mice over-expressing human wild type or familial AD mutant APP. (A) Western blot of APP and PrP^C^ (antibody 6D11) in I5 (n = 3) and J20 (n = 2) transgenic, and age-matched non-transgenic control, mouse brain homogenates, with membrane re-probing for β-actin. Approximate molecular weights (kDa) are indicated. (B) Quantification of APP and PrP^C^ protein levels expressed relative to the control mice (dashed line). Error bars represent ± SD. Statistical analysis by unpaired t-test, **p<0.01, n.s. not significant.

To further examine the effect of APP over-expression on PrP^C^ levels, two transgenic mouse models were investigated. PrP^C^ and APP protein levels were evaluated in brain homogenates from I5 mice which over-express wild type human APP, J20 mice which over-express human APP containing the Swedish/Indiana familial AD mutations [23] and non-transgenic matched genetic background control mice (Fig. 3A and B). Despite a significant 2.8 -fold increase in APP in the transgenic I5 mice, as compared to the non-transgenic mice, there was no difference in brain PrP^C^ levels. Analysis of the J20 mice, although only involving two animals, reinforced this conclusion. Collectively these over-expression experiments indicate that control of PrP^C^ expression does not appear to involve AICD in either cell-based or transgenic animal paradigms.

### Reduction of AICD production through APP gene silencing or γ-secretase inhibition does not alter expression of endogenous PrP^C^

**Figure 4:**
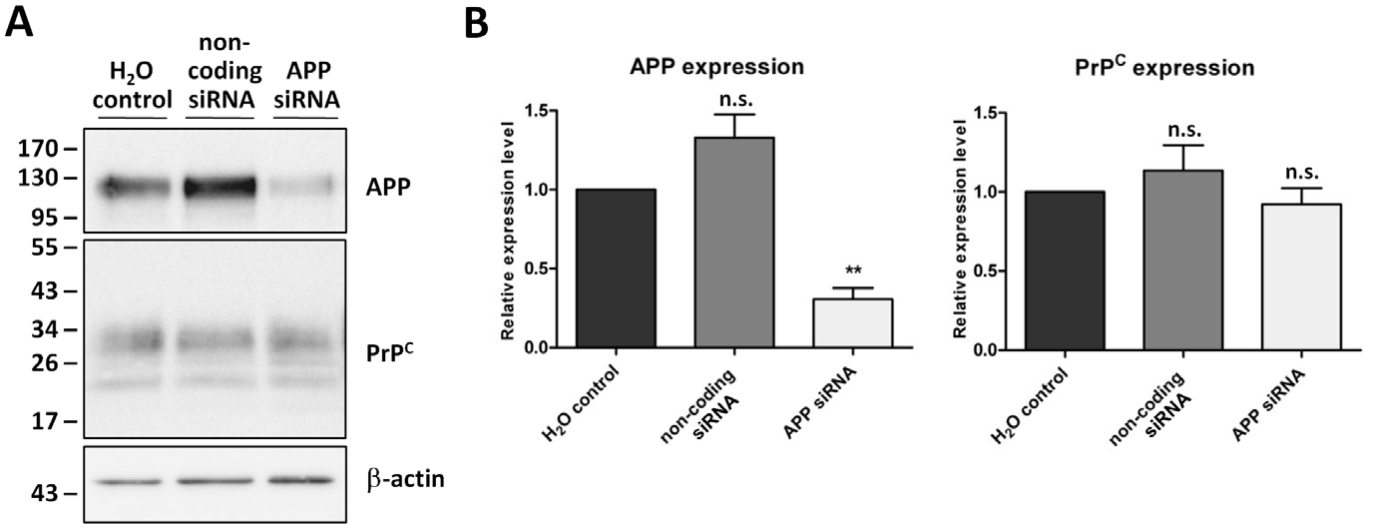
Knockdown of APP expression in N2a cells has no effect on PrP^C^ protein levels. (A) Representative western blots of APP and PrP^C^ (antibody 6H4) in N2a cells treated with APP directed siRNA, non-coding control siRNA, and a no RNA transfection control (H_2_O control), and subsequent β-actin staining. Approximate molecular weights (kDa) are indicated. (B) Quantification of APP and PrP^C^ protein levels expressed relative to the H_2_O control cells. Data from 3 independent experiments. Statistical analysis by one way ANOVA with Dunnett's post test comparison to the H_2_O control cells, **p<0.01, n.s. not significant.

In light of the above results, we considered whether the level of AICD required to regulate PrP^C^ expression in the cell lines or the transgenic mice were already maximal from the endogenous APP, such that the AICD produced from the over-expressed APP was not having any additional affect on PrP^C^ expression. Thus we sought to investigate a possible role for endogenous AICD in the control of PrP^C^ expression. First, to reduce endogenous APP levels and thereby remove the substrate for AICD production, N2a cells were treated with siRNA against murine APP. Cells were harvested, lysed and PrP^C^ and APP levels measured by western blotting (Fig. 4A and B). After directed siRNA treatment there was a significant 70% decrease in total APP levels (endogenous AICD level is below the limits of detection by immunoblot; data not shown). However, the amount of PrP^C^ remained unchanged following siRNA knockdown of endogenous APP.

**Figure 5:**
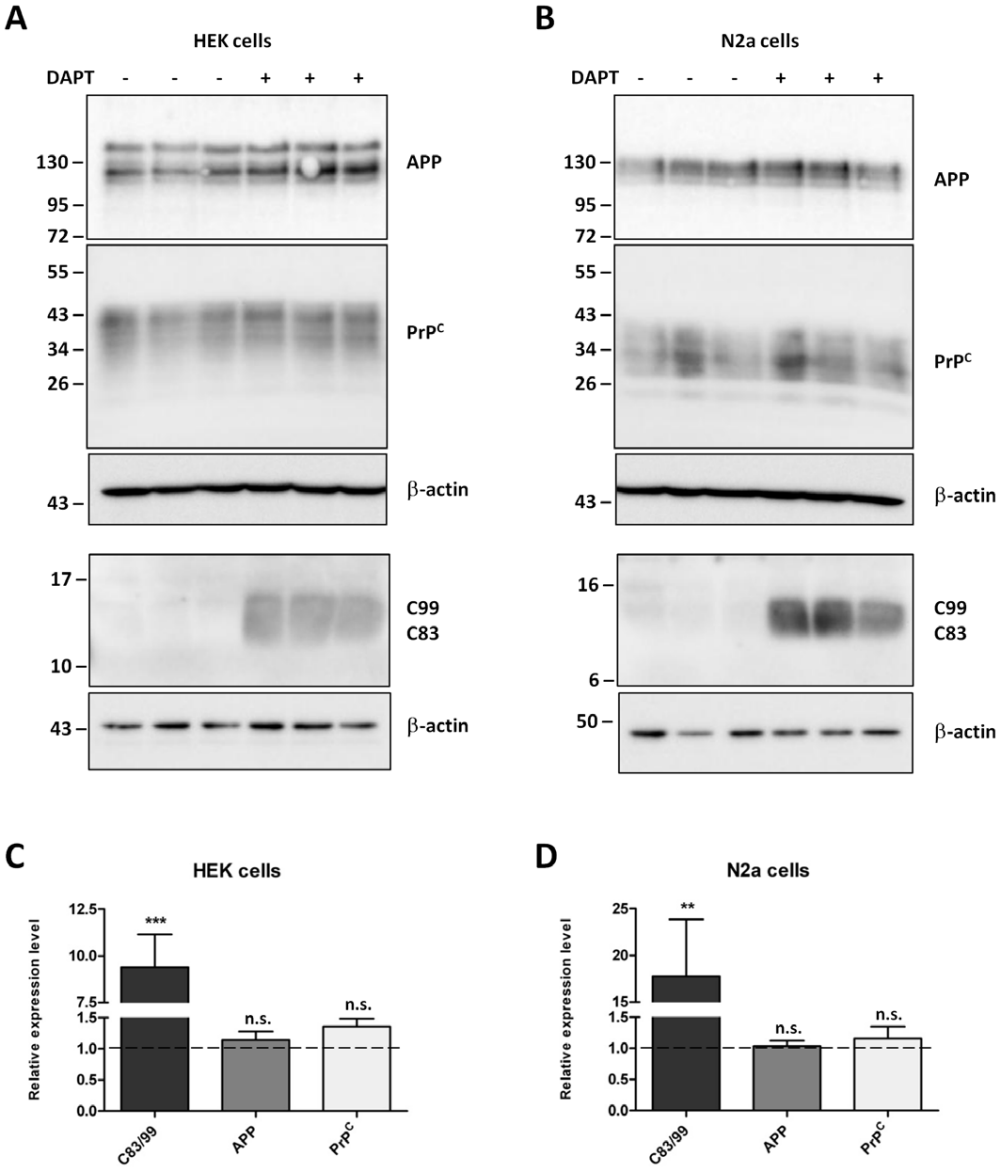
Inhibition of γ-secretase activity does not alter the expression of PrP^C^. Representative western blots showing total cell-associated APP, PrP^C^ (antibody 6H4) and C-terminal APP fragments (C83/99) in control (−) and DAPT treated (+) HEK (A) and N2a (B) cells, with membrane re-probing for β-actin. Approximate molecular weights (kDa) are indicated. Quantification of C83/99, APP and PrP^C^ protein levels in DAPT treated HEK (C) and N2a (D) cells, expressed relative to control cells (dashed line). Data from 3 (HEK) or 4 (N2a) independent experiments. Statistical analysis by one way ANOVA with Dunnett's post test comparison to the control cells, ***p<0.001, **p<0.01, n.s. not significant.

In order to test further for a possible involvement of endogenous AICD in controlling PrP^C^ expression, both HEK and N2a cells were treated with DAPT, a cell permeable γ-secretase inhibitor. Again, whole cell lysates were assessed for PrP^C^ and APP expression, as well as the levels of C83 and C99, by western blotting (Fig. 5A and B). Although DAPT treatment inhibited γ-secretase activity, as shown by the significantly increased C83 and C99 levels (9.4-fold in the HEK cells and 17.8-fold in the N2a cells), there was no difference in endogenous PrP^C^ protein levels in the DAPT treated cells as compared to the untreated cells (Fig. 5C and D). Together these results indicate that in both a neuronal and a non-neuronal cell line, endogenous AICD is also not involved in the control of PrP^C^ protein expression.

## Discussion

Similarities in the pathogenesis of the protein-misfolding neurodegenerative illnesses, especially AD and prion diseases, and possible connections between these diseases have long been contemplated [1], [2], [3], [4]. Elucidation of any functional links between these diseases is an important research goal, with determination of the most appropriate protein or process to target for development of therapeutics being paramount. Links in the pathologies of AD and prion diseases have been determined, with various reports of AD features in prion disease brains [24], [25], [26], and PrP^C^ localised in Aβ plaques in AD brain [27], [28]. In addition, a polymorphism at codon 129 of the prion protein gene, known to influence susceptibility to sporadic and iatrogenic human prion disease [29], [30], may also influence susceptibility and the pathophysiology of AD [31], [32], [33]. Interestingly there is some indication of a more direct interaction between Aβ and PrP^Sc^, with the finding of an acceleration and exacerbation of both AD and prion disease pathologies in animals engineered to have both of these diseases, and enhanced protein misfolding due to cross-seeding events stimulating oligomerization *in vitro* [34]. This propensity for cross-seeding highlights the importance for a more complete understanding of interactions between these key proteins and any resultant downstream consequences.

Recent studies have provided evidence of direct interactions between the proteins central to AD and prion diseases. Various studies have determined that the cellular prion protein can act as a receptor for Aβ, with Aβ oligomers binding to PrP^C^ with high affinity, although there are conflicting views as to the physiological significance of this binding. Some results suggest that Aβ synaptic toxicity is mediated through its binding to PrP^C^ [7], [11], [12], which specifically impacts on spatial learning and memory *in vivo* [35], whereas others have reported that Aβ oligomer neurotoxicity occurs independently [8], [9]. Confounding the relationship between these key proteins, and in apparent contrast to PrP^C^ mediating Aβ neurotoxicitiy, PrP^C^ has been shown to decrease production of Aβ from wild type APP through its interaction with the β-secretase BACE1 [5]. This interaction, mapped to the BACE1 pro-domain, leads to slowed BACE1 trafficking following exit from the ER, thereby increasing its localization in the trans-Golgi network and reducing levels at the cell surface and consequently in endosomes where APP β-cleavage occurs [6]. Importantly, these studies also ascertained links in the pathology of AD and prion diseases. It was found that human prion disease-associated mutations in PrP^C^ did not inhibit BACE1, and scrapie infected mice brains contained dramatically higher Aβ levels [5], suggesting a loss of PrP^C^ function perhaps as a result of PrP^C^-PrP^Sc^ conversion during prion disease progression.

PrP^C^ may be a key therapeutic target for sporadic AD, and the recent report that PrP^C^ expression was controlled by AICD in a γ-secretase dependent manner [22] presented a potential avenue for achieving this. Further, a possible feedback model reconciling the control of APP processing and PrP^C^ expression in both normal conditions and in the presence of increased Aβ such as that seen in AD was proposed [36]. Therefore our study was carried out to further understand the relationship between AICD production and PrP^C^ expression. However, utilizing a range of experimental approaches we found no evidence for AICD involvement in PrP^C^ expression. This is despite using a cellular system (N2a cells expressing APP_695_) in which we have proven that AICD is transcriptionally active [19]. If AICD is involved in regulating the transcription of PrP^C^, then the mechanism underlying this is more complex than that involved in regulating the expression of neprilysin and is not readily reproduced in cultured cells or transgenic mice over-expressing human APP. Our findings have implications for the continued investigation and design of possible AD therapeutics.

## Materials and Methods

### Ethics statement

All experimental procedures performed on mice were approved by the Roslin Institute (University of Edinburgh) Ethical Review Process Committee and carried out under the UK Home Office License 60/3478. Human embryonic kidney (HEK) cells were obtained from the European Collection of Cell Cultures and murine neuroblastoma (N2a) cells were obtained from Dr Lehmann, Université Montpellier, France [37].

### Cell culture

HEK cells and N2a cells stably over-expressing the human APP isoforms (APP_695_, APP_751_ and APP_770_), alongside the vector-only (Hyg), were generated by electroporation as described previously [19]. Cells were maintained in Dulbecco's modified Eagle's medium (DMEM; Lonza, Basel, Switzerland) containing 10% (v/v) fetal bovine serum (FBS; Biosera, East Sussex, UK) and 1% penicillin/streptomycin (Lonza), in a humidified incubator at 37°C, 5% CO_2_.

### APP gene silencing

To ablate endogenous APP expression in the N2a cells, cells were grown to 80% confluency in growth medium prior to treatment with 50 nM final concentration of murine APP directed siRNA, non-coding siRNA or siRNA-free controls following the manufacturer's instructions (Thermo Fisher Scientific, Lafayette, CO, USA). Briefly, sub-confluent cell monolayers were washed gently with OptiMEM (GIBCO, Invitrogen, Glasgow, UK), before further incubation in OptiMEM for approximately 30 min (37°C, 5% CO_2_) during siRNA preparation. A 10 µM siRNA solution in 1× siRNA buffer of either murine APP directed siRNA (ON-TARGETplus SMARTpool) or non-coding siRNA control (ON-TARGETplus Non-targeting pool) was prepared, and diluted to 1 µM in OptiMEM. For the RNA-free control, sterile RNase-free water was diluted 1∶10 in OptiMEM. DharmaFECT Transfection Reagent-1 was diluted 1∶40 in OptiMEM, mixed gently and incubated for 5 min at room temperature. Equal volumes of the diluted siRNA/control and DharmaFECT solutions were then mixed and incubated for 20 min at room temperature, prior to the addition of 4× volumes of OptiMEM containing 10% (v/v) FBS. The OptiMEM was then removed from the cells, and replaced with the OptiMEM/FBS/siRNA complexes (or control) for 72 h.

### Inhibition of γ-secretase

To inhibit endogenous γ-secretase activity, HEK or N2a cells were grown to 90–95% confluency prior to treatment. The cell monolayer was then washed twice with PBS prior to incubating the cells in serum-free OptiMEM containing a final concentration 10 µM N-[N-(3,5-difluorophenacetyl)-L-alanyl]-S-phenylglycine t-butyl ester (DAPT; Sigma-Aldrich, Dorset, UK), or an equal volume of dimethyl sulfoxide (DMSO, as the control) for 24 h.

### Cell lysis, SDS-PAGE and immunoblotting

When confluent and/or after appropriate treatments as described above, cells were washed twice in phosphate-buffered saline (PBS with Ca^2+^ and Mg^2+^; Lonza), harvested by scraping into PBS, and pelleted at 500× g for 3 min. Cell pellets were lysed for 30 min on ice in cold lysis buffer (25 mM Tris/HCl, pH7.5, 150 mM NaCl, 5 mM EDTA, 1% (v/v) Triton X-100) containing Complete™ protease inhibitor cocktail (Roche, West Sussex, UK), prior to centrifugation for 5 min at 1000× g. Post-nuclear supernatants were assessed for total protein content using a bicinchoninic acid protein assay (Sigma-Aldrich). Cell lysate containing 50 µg total protein was resolved on 7–17% (APP and PrP^C^) or 16.5% (C83/99) polyacrylamide SDS gel, then electrotransferred to Hybond-P polyvinylidene difluoride membrane (PVDF; Amersham Life Sciences, Buckinghamshire, UK). PVDF membranes were blocked for 1–2 h at room temperature in PBS containing 0.1% Tween-20 (PBST) and 5% (w/v) skimmed milk powder, prior to incubation with primary antibody overnight at 4°C. For detection of APP the membrane was incubated with the monoclonal antibody 22C11 (Millipore, Billerica, MA, USA), and for detection of PrP^C^ the membrane was incubated with either the monoclonal antibody 3F4 (Covance, Princeton, NJ, USA), 6D11 (Eurogentec Ltd, Hampshire, UK) or 6H4 (Prionics, Zurich, Switzerland), as indicated in the figure legends. For detection of C83/99, a polyclonal anti-C-terminal APP antibody A8717 (Sigma-Aldrich) was used. After washing off non-specifically bound primary antibody with PBST, membranes were incubated in peroxidase-conjugated rabbit-anti-mouse or goat-anti-rabbit secondary antibodies (Sigma-Aldrich), before further washes with PBST and detection using enhanced chemiluminescence (Pierce ECL substrate; Thermo Fischer Scientific). To assess and correct for protein loading, membranes were stripped at low pH (1% (v/v) aqueous HCl) for approximately 30 min, re-blocked and probed with an anti-β-actin antibody (clone AC-15, Sigma-Aldrich) and the secondary antibody described above. All chemiluminescent images were captured by a Fujifilm LAS-3000 Intelligent Dark Box.

### Transgenic mice and tissue homogenisation

Animals were obtained from The Jackson Laboratory (Bar Harbor, ME, USA), and all care was carried out in strict accordance with institutional guidelines. Transgenic I5 mice (Line B6.Cg-Tg(PDGFB-APP)5 Lms/J, stock number 004662), which over-express wild type human APP, and J20 mice (Line B6.Cg-Tg(PDGFB-APPSwInd)20 Lms/2J, stock number 006293), which over-express human APP containing the Swedish (K670N/M671L) and Indiana (V717F) familial AD mutations [23], were crossed with inbred 129P2 mice, and genotyped to confirm the *APP* gene sequence. Brain hemispheres from the I5 (8 weeks old), J20 (5–9 weeks old) and age-matched non-transgenic littermate controls were homogenized in 2% (w/v) SDS solution containing protease inhibitors, and homogenates centrifuged at 100,000× g for 1 h at 4°C. The resultant supernatant was assayed for total protein and assessed for PrP^C^ and APP protein by SDS-PAGE and western blotting as described above for cell lysates.

### Densitometry and statistical analysis

Quantification and densitometric analyses were carried out using Image J v1.42q. Within each experiment, data was normalised to β-actin, and expressed relative to the control samples. Statistical analyses were performed in GraphPad Prism v5.03. All quantitative data are expressed as the mean ± SEM, unless stated otherwise.
